# The altering in sensory sensitivity: a current issue of female breast surgery

**DOI:** 10.7150/ijms.71913

**Published:** 2022-05-16

**Authors:** Tong Zhu, Yi Jiang, Ting Liu, Jinqi Xue, Nan Niu, Jiawen Bu, Mingxin Liu, Caigang Liu, Xudong Zhu, Xi Gu

**Affiliations:** 1 Department of Oncology, Shengjing Hospital of China Medical University, Shenyang, Liaoning 110004, China; 2 Department of Laboratory, Shenyang Medin Women's and Children's Hospital, Shenyang, Liaoning 110032, China; 3 Department of General Surgery, Cancer Hospital of China Medical University, Liaoning Cancer Hospital and Institute, Shenyang, Liaoning 110042, China

**Keywords:** breast, breast surgery, breast skin sensitivity

## Abstract

Breast surgery is an important treatment for women with malignant breast diseases. In addition to breast appearance, the integrity of breast function is increasing in patients with breast diseases. As the basis of breast physiological function, breast skin sensitivity is important to the quality of life of patients after surgery. Breast skin sensitivity gives the patient a “real” breast feeling. The sensory recovery after breast surgery has also become one of the important goals of breast surgery. In this review, we aim to discuss the research progress on recovery of breast skin sensitivity after different treatment modalities for breast disease.

## Introduction

The incidence of breast diseases in women is increasing daily. Among female malignant tumors, breast tumors have the highest incidence and endanger women's health. Surgery is the main treatment for breast disease. Meanwhile, with the advances in medical treatment of tumors, the postoperative survival time of malignant breast tumors has improved, and the 5-year survival rate is up to 90% 1. This survival prolongation has led to an increase in the requirements to maintain the patients' quality of life postoperatively. Apart from protection of the breasts' beautiful appearance, an increasing number of women pay attention to the physiological function of the breast during the treatment of benign and malignant breast diseases. The sensitivity of the breast skin is fundamental to the physiological function of the breast, not only to avoid accidents such as burns, but also to give women a “real” breast sensation. However, different breast surgeries have different effects on breast sensation, and the recovery of breast sensation after surgery varies. In this review, we aim to discuss the research progress on recovery of breast skin sensitivity after different treatment modalities for breast disease.

## 1. Neuroanatomy of the breast

The upper and lower borders of the normal adult female breast lie on the second and sixth ribs, respectively. The inner border is the edge of the sternum, and the outer border is the midline of the axilla. The regional nerves include the breast branch of the supraclavicular nerve, medial branch of the lateral cutaneous branch of the intercostal nerve 2-6, and lateral branch of the anterior cutaneous nerve 2. The breast branch of the supraclavicular nerve joins the anterior branch of the cervical nerve 3-4 through the cervical plexus and is responsible for only a small part of the sensory function in the upper part of the breast. Most of breast skin sensitivity depends on the medial branch of the lateral cutaneous branch of the intercostal nerve and the medial branch of the anterior cutaneous nerve. The medial branches of the lateral cutaneous branch of the intercostal nerve extend from the anterior serratus anterior on the axilla, and the lateral branches of the anterior cutaneous branch of the intercostal nerve pass through the parasternal pectoralis major to the midclavicular line 3. The lateral cutaneous branch innervates mainly the lateral region of the breast, and the anterior cutaneous branch is responsible for the medial region of the breast; therefore, the scope of dominance of the two also shows a certain degree of complementarity. Similarly, although the cutaneous branches of the intercostal nerves are distributed in segments and are parallel to the ribs, there is a cross distribution of small branches between the two adjacent cutaneous branches, which can reach the upper and lower intercostal areas. Several small branches of the second intercostal nerve are mainly distributed in the skin of the upper part of the breast and hardly reach the areola area. The branches of the third intercostal nerve are also distributed in the upper part of the breast, but few small branches reach the areola. The 4^th^ intercostal nerve is relatively large and mainly distributed in the central area of the breast. The 5^th^ and 6^th^ intercostobrachial nerves correspond to the 2^nd^ and 3^rd^ intercostobrachial nerves, respectively, and are responsible for the cutaneous sensory function of the lower part of the breast 4-6.

The nipple-areola complex is mainly innervated by the 3rd-5th intercostal nerves, with the 4th intercostal nerve being the most important 7. The anterior cutaneous branch of the intercostal nerve passes through the point of penetration and travels through the subcutaneous fat layer of the breast, then gradually towards the nipple-areola area. The most important lateral cutaneous branch of the 4th intercostal nerve passes through the intersection of the lateral border of the pectoralis major muscle and the 4th intercostal space, and then divides into two deep and superficial branches to reach the nipple areola area 8. The deep branch passes through the deep mammary tissue to reach the nipple areola, while the superficial branch divides into five branches that travel through the superficial mammary tissue to reach the nipple areola. One central branch reaches the nipple, two upper branches reach the upper part of the areola, and two lower branches reach the lower part of the areola 4. Thus, the distribution of nerves in the breast and nipple-areola area is incrementally distributed in a centripetal fashion, crossed and without clear boundaries. The external lower quadrant of the nipple areola region is the most sensitive, followed by the internal upper, internal lower and external upper quadrants.

Skin sensitivity differs in different parts of the breast. Tactile sensitivity is highest in the nipple, followed by the upper quadrant of the breast, areola, and lower quadrant of the breast. Regarding vibration sensitivity, the most sensitive is the nipple, followed by the areola, skin of the lower quadrant, and skin of the upper quadrant. The pressure threshold of the skin of the breast is proportional to the breast volume, that is, the larger the breast volume, the less sensitive the breast 2, 9. Tactile sensitivity decreases with an increasing in the size and sag of breast.

## 2. Criteria for evaluating the neurologic function of the breast

The evaluation and testing criteria for breast nerve function are divided into subjective or objective. Subjective evaluation is based on patients' self-evaluations after breast surgery. Generally, the BREAST-Q scale is used in clinical settings and may be adjusted 10, 11. Objective physical stimulation tests are used for the objective sensory evaluation system; these tests are based on the patients' responses. For example, the acupuncture is used to measure pain sensitivity, the cotton is used to measure the sensitivity of touch and pressure, heat and cold are used to measure temperature sensitivity, and the tuning fork is used to measure vibration sensitivity. Common objective sensory evaluation methods include the Semmes-Weinstein monofilament examination and von Frey test for quantifying the skin pressure threshold 12, probabilistic species sensitivity distribution (PSSD) method for continuously measuring pressure sensitivity in the skin using a computerized system; the less commonly methods, such as Weber static two-point discrimination test, Dellon dynamic two-point discrimination test 13-15. Relevant studies that used common objective measures as well as subjective measures are organized in Table 1.

After nerve damage during breast surgery, skin sensitivity is restored in the following order: pain, pressure, temperature, and two-point discrimination 17. Currently, BMRC, the mackinnon-dellon scale, and the modified mackinnon-dellon scale are used to evaluate the skin sensory function after breast reconstruction; BMRC is the most used. Based on these methods, breast sensitivity is measured in five, six, and nine regions, respectively 40, 45, 61. In addition, the timing for breast sensory evaluation after breast reconstruction is controversial. Mageraki et al. suggested that that skin sensitivity returns only 18-24 months after breast reconstruction; therefore, it is recommended that the evaluation of postoperative skin sensitivity should be performed within this interval 62.

## 3. The effect of surgery of different benign breast diseases on breast skin sensitivity

### 3.1 The effect of benign breast tumor operation on breast skin sensitivity

Benign breast tumors are the commonest breast diseases in women, especially young ones. Surgery is the main treatment 63. Currently, radial incisions, Langer's line incisions, circum-areolar margin incisions and intra-areolar incisions are surgical incisions used during benign breast tumor surgeries. When choosing incisions, clinicians mainly consider whether the tumor can be removed easily and completely, the incision is concealed, and the incision ensures esthetics after healing. However, little consideration is given to sensitivity loss after local skin injury as it is of less concern to some young women. This suggests that attention should be paid not only to the above factors, but also to the orientation of the cutaneous sensory nerves at the incision site of the breast. Furthermore, sensory abnormalities of the breast skin should be minimized by avoiding nerve damage. A researcher compared the areolar edge incision with the intra-areolar incision used for breast fibroadenoma resection. They observed that patients who chose the intra-areolar incision had a good sensory recovery of the areolar skin 64. Other studies have shown that the medial incision of the areola has a significantly lower impact on the nerves than the lower incision of the areola 65. Several researchers had controversial findings: they observed that even with a circum-areolar incision at the 4 o'clock position of the left breast or the 8 o'clock position of the right breast, which is most likely to favor an injury of the lateral cutaneous branch of the 4^th^ intercostal nerve, there was no significant difference in the recovery time of the nipple and areola when compared with other incisions. The location and length of the circumferential incision did not affect the recovery time of the nipple and areola 66.

### 3.2 Effect of breast reduction and plasty on breast skin sensitivity

Breast reduction surgery can relieve the pain of patients with breast hypertrophy. It can also be used for patients with breast cancer undergoing breast symmetry surgery. Breast-conserving surgery may be used in the middle of breast reduction surgery for some patients with chronic mastitis and breast cancer. The sensitivity of the nipple areola and breast skin decreases within 3 months postoperatively, but the tactile sensitivity of the breast is similar to that 1 year before the surgery 67, 68. Sensitivity returns faster in the nipple than in the areola, and faster in the upper quadrant faster than in the lower quadrant 69. Sensitivity to touch returns 2 weeks postoperatively and continues to increase within 1 year. Moreover, nipple erection is not affected 70. Using the nipple-free vertical technique, some researchers observed that breast skin sensitivity changed a little after surgery and recovered earlier. Compared with the sensitivity after use of the lower pedicle technique 71, sensitivity decreased significantly. Other studies have shown that in patients with a modified double-loop reduction of large breasts, the sensory recovery of the nipple areola was good, and the lactation function was retained 72. In addition, the volume of excised tissue is also an important factor for skin sensory function. Most scholars believe that there is a positive correlation of amount of excised tissue with the number of severed nerves and risk of papillary and areolar sensory injury 73.

### 3.3 Effect of breast augmentation on breast skin sensitivity

Breast augmentation is performed by the implantation or self-fat tissue. It helps to increase breast volume and improve women's body shape. It is good procedure for women with the poor development of breast. Implantation is the most common method. Studies have shown that skin sensitivity in the breasts after implants is lost for a short period, but it returns to its pre-operative state approximately 12 weeks postoperatively. The area of decreased sensitivity after implantation is directly related to the surgical approach. The sensitivity of the lower outer quadrant of the breast decreases with the use of a submammary incision^6^, and the sensitivity of the nipple-areola complex decreases significantly with the use of a periareolar incision 6, 74. The larger the implant size, the less sensitive the nipple-areola complex 75.

## 4. The effect of different breast cancer operations on breast skin sensitivity

### 4.1 Sensitivity of chest wall skin after no-skin sparing mastectomy

Modified radical mastectomy is one of the most widely used surgical methods for breast cancer because of the total removal of breast tissue and glands, absence of the breast skin, and complete loss of breast sensitivity. After no-skin sparing mastectomy, breast sensitivity, including sexual sensitivity is almost completely lost. Importantly, the ability of the breast skin to protect sexual sensitivity is lost, and patients can not feel the stimulation of skin lesions in the primary breast area, which greatly affects their quality of life postoperatively 6, 21, 76.

### 4.2 Effect of nipple and areola-sparing surgery on breast skin sensitivity

Nipple and areola‐sparing mastectomy (NSM) are the basis of breast reconstruction for breast cancer. The breast skin is well-preserved, which provides the basic condition for the recovery of the breast nerve function. The selection of the incision position is the main factor for postoperative sensory recovery after NSM. The objective sensitivity of the nipple and areola decreases significantly after surgery with the use of areolar and sub-mammary incisions; it is possibly due to the destruction of the cutaneous branch of the 4^th^ intercostal nerve 77, 78. In addition, a small and short incision during NSM to protect the breast innervation system contributes to postoperative breast sensitivity, and these patients with breast cancer can regain papillary erection 6-12 months postoperatively 44. Other studies have shown that using an endoscopic surgery for breast cancer with sparing the nipple and areola avoids incisions around the nipple and areola complex, which greatly reduces the likelihood of sensory impairment of the nipple and areola complex. Compared to patients who undergo NSM, there is a significant reduction or impairment in pressure, temperature, and vibration perception in patients who undergo endoscopic surgery 79.

### 4.3 Breast reconstruction surgery

#### 4.3.1 Effect of autologous tissue reconstruction on breast skin sensitivity

Breast reconstruction with breast autografts gives a natural feeling, is long-lasting, and patient satisfaction is high. Currently, the most used autologous tissue flaps for breast reconstruction are deep inferior epigastric perforator (DIEP), the latissimus dorsi (LD) flap and transverse rectus abdominis muscle (TRAM) flap. Breast skin sensitivity is best after the use of DIEP, followed by LD and TRAM 33. Table 2 summarizes some studies that compared results with and without nerve grafts.

The DIEP flap is the gold standard for autologous breast reconstruction because of its good shaping ability and minimal donor site damage. The DIEP flap is constituted of the abdominal wall skin and subcutaneous fat flap. The neurotized flap contains sensory nerves. Even without nerve, approximately 70% of patients show signs of reinnervation after reconstruction. The degree of skin sensitivity recovery 6-12 months postoperatively and the recovery time are proportional to the degree of recovery 22, 53, 82, 83. Most scholars believe that the sensitivity of the lower part of the breast recovers better than that of the nipple area and upper part of the breast, and that tactile sensitivity recovers better than other sensitivities. Regarding DIEP reconstruction with nerve anastomosis, breast sensitivity is recovered early and good, and 30% of patients have sexual sensitivity after DIEP reconstruction, which greatly improves their quality of life and satisfaction 17, 29, 84.

The LD flap is the common flap for breast reconstruction because of its good strain capacity and low surgical difficulty 85. However, the LD flap is a musculocutaneous flap with a predominant motor innervation than sensory innervation. Therefore, compared to the sensitivity of DIEP reconstruction, the sensitivity of LD flap reconstruction is worse. Although the effect can be improved after nerve anastomosis, there are still few patients who cannot restore skin sensitivity, and most patients have a complete loss of sexual sensitivity 86, 87.

The TRAM flap is also a musculocutaneous flap located near the breast. TRAM breast reconstruction is more effective in patients with nerve than in those without 41. Neurotized TRAM reconstruction begins approximately 6 months postoperatively and is complete at approximately 15-18 months after surgery. Meanwhile, non-neurotized TRAM reconstruction ensures little sensory recovery in the first 10 months and takes a year for recovery to begin 87.

#### 4.3.2 Effect of implant reconstruction on breast skin sensitivity

Breast reconstruction with implants after breast cancer surgery does not require damage to other parts of the autologous tissues. The operation is relatively simple, and the reconstructed breast has a beautiful appearance. The use of implants is currently the most used breast reconstruction method for breast cancer; however, there are few studies on breast skin sensitivity after breast reconstruction with implants. Some studies have reported decreased sensitivity in the breast, especially in the region of the nipple and areola, after reconstruction with a stage I breast implant 88. A study reported that that the sensitivity of the skin in the four quadrants of the breast after implant reconstruction was lower than that after DIEP flap reconstruction 62. It has also been reported that implants are usually placed behind the pectoralis major during implantology reconstruction, and that the use of Tiloop patches in combination with implantology is superior to conventional implantology 89. From the findings of the existing studies, it seems that breast sensitivity after reconstruction with implants is weaker than that after reconstruction with autologous tissues. This may be due to the fact that the implant is placed behind the pectoralis major and blocks the innervation of the nerves from the pectoralis major—the compression of the implants leads to the increase in skin tension, which affects the regeneration of the skin nerves and reduces breast skin sensitivity.

#### 4.3.3 Effects of immediate and delayed reconstruction on breast skin sensitivity

The decision to perform immediate and delayed reconstruction depends on the clinical stage of breast cancer, the need for postoperative radiotherapy, and patient's personal decision. Studies have shown that immediate reconstruction of the DIEP flap or TRAM flap after skin-sparing mastectomy is faster and ensures a better sensory recovery of the region from the nipple areola to the skin of the whole breast than delayed reconstruction 37. Immediate reconstruction is also better than staged delayed reconstruction when the implant is selected 62. Although few studies have shown no significant difference in sensitivity recovery between immediate and delayed reconstruction 41, the overall analysis suggests that immediate reconstruction is more effective than delayed reconstruction of the breast skin.

## 5. Effect of radiotherapy on breast skin sensation in breast cancer patients

Radiotherapy plays an important role in the comprehensive treatment of breast cancer. For high-risk breast cancer patients with positive lymph nodes, whether they choose mastectomy or breast reconstruction, postoperative adjuvant radiotherapy not only improves local control of the tumor, but also benefits long-term survival 90. However, radiotherapy can cause numerous adverse effects such as skin irritation, radiation dermatitis, and chest wall tenderness. Almost all (99.8%) nipple-areola preserving mastectomies (NSM) have side effects after radiotherapy, with skin irritation and thickening and chest wall tenderness being the most common; about 94.3% of mastectomy patients (CM) experience complications such as loss or alteration of nipple or breast sensation and chest wall tenderness, but rarely do they experience persistent pain for longer than 6 months^91^. Moreover, pain associated with breast radiotherapy generally peaks 1 week after the completion of radiotherapy 92. Moreover, with irradiation, non-neurotized DIEP flap skin had better sensation recovery than did skin over implants. However, without irradiation, skin overlying implants is associated with better sensation recovery than non-neurotized DIEP flap skin 62. Another study further verified that intraoperative radiotherapy does not have a greater improvement in the occurrence of pain compared to external breast radiotherapy 93. In conclusion, radiotherapy can cause many adverse effects, the most severe of which is pain in terms of skin sensation.

## 6. Summary and outlook

The breast is a key organ for women, and the recovery of breast sensitivity after breast surgery is of growing interest. This has led to increasing research in the protection of breast skin sensitivity, breast neuroanatomy, and the characteristics of different treatment modalities for breast disease. Currently, there is no unified system for evaluating all breast sensitivity functions after breast surgery. The recovery of breast skin sensation varies with different treatment modalities for breast disease and is influenced by the choice of surgical incision, extent of excision, surgical procedure, etc. The mechanisms of sensory self-repair and nerve regeneration in the breast skin after breast surgery are unclear. Nerve anastomosis in breast reconstruction can effectively improve breast skin sensitivity, but the methods of nerve reconstruction still require further research. With the development of medical technology, it is anticipated that women with breast diseases can be provided the “perfect” breasts, which have both esthetics and good functions.

## Figures and Tables

**Table 1:** Common methods of sensory assessment

Methods	Author/Year			
S-W von-frey	Beugels et al./2021^16^	Beugels et al./2019^17^	Verscher et al./2016^18^	Yap et al./2005^19^
	Zacchê et al./2021^20^	Bijkerk et al./2019^21^	Klasson et al./2014^22^	Schlenz et al./2005^23^
	Momeni et al./2021^24^	Pek et al./2018^25^	Godbout et al./2013^26^	Hefter et al./2003^27^
	Tevlin et al./2021^28^	Cornelissen et al./2018^29^	Chiari Jr et al./2012^30^	Hamdi et al./2003^31^
	Beugels et a/2021^32^	Cornelissen et al./2018^33^	Mori et al./2011^34^	Mofid et al./2002^35^
	Sihol et al./2021^36^	Heine et al./2017^37^	Puonti et al./2011^38^	Yano et al./2002^39^
	Bijkerk et al./2020^40^	Puonti et al./2017^41^	Tindholdt et al./2008^42^	Blondeel et al./1999^43^
	Dogan et al./2020^44^	Dossett et al./2016^45^	Pitanguy et al./2007^46^	Edsander-Nord et al./1999^47^
	Zacchê et al./2020^48^	Brown et al./2016^49^	Temple et al./2006^50^	Place et al./1997^51^
	Masgoret et al./2020^52^	Stromps et al./2016^53^		
PSSD	Djohan et al./2020^54^			
	Rodriguez-Unda et al./2017^54^			
	Spiegel et al./2013^55^			
	Longo et al./2013^56^			
	Santanelli et al./2011^57^			
Breast-Q	Dejean et al./2021^58^			
	Opsomer et al./2020^59^			
	Cornelissen et al./2018^29^			
	Verschuer et al./2016^18^			
	Temple et al./2009^60^			

S-W: Semmes-Weinstein monofilament PSSD: probabilistic species sensitivity distribution

**Table 2:** Summary of different surgical technique of breast reconstruction

Author	Year	Surgical technique	Assessmont methods	Donor nerve	Recipient Nerve	Results with Neurotization
Beugels et al. 32	2021	LTP	S-W	LFCN	ACB of ICN	better sensory recovery
Bijkerk et al. 40	2020	DIEP/LTP	S-W	ICN 10^th^ -12^th^ thoracic nerve /LFCN or ACB of ACFN	ACB of 2^nd^ -3^rd^ ICN	better sensation
Beugels et al. 17	2019	DIEP	S-W	a cutaneous branch of ICN 10^th^-12^th^ thoracic nerve	ACB of ICN	better sensory recovery
Spiegel et al. 55	2013	DIEP	PSSD	ACB of ICN 11^th^-12^th^ thoracic nerve	ACB of 3^rd^ ICN	increasing sensory recovery
Magarakis et al. 62	2013	DIEP	PSSD	ACB of ICN 10^th^ -12^th^ thoracic nerve	AB-LCB of 4^th^ -5^th^ ICN	better sensory
Puonti et al. 38	2011	TRAM	S-W	ACB of ICN 10^th^ -12^th^ thoracic nerve	the thoracodorsal nerve the intercostobrachial nerve intercostal nerve	improved sensory recovery
Mori et al. 34	2011	TRAM	S-W	ACB of ICN 10^th^ -12^th^ thoracic nerve	AB-LCB 4th ICN	More sensitive sensory
Temple et al. 60	2009	TRAM	BREAST-Q	ICN 10^th^ thoracic nerve	4^th^ ICN	Improves sensibility of sensory and quality of life.
Temple et al. 50	2006	TRAM	S-W	10^th^ thoracic nerve	LCB of 4^th^ ICN	provides improved sensation
Yap et al. 19	2005	TRAM	S-W	thoracoabdominal nerve	LCB of 4^th^ -5^th^ ICN	better sensory recovery
Isenberg et al. 80	2004	TRAM/LD	S-W	ACB of ICN 11^th^ thoracic nerve / thoracodorsal nerve	AB-LCB of 4^th^ ICN	sensory levels approached but did not equal contralateral healthy breast
Yano et al. 39	2002	LD	S-W	LCB of 7^th^ thoracic nerve	LCB of 4^th^ ICN	better sensory recovery
Isenberg et al. 81	2002	TRAM	S-W	ACB of ICN 11^th^ thoracic nerve	ACB of ICN T11	More rapid and improved
Blondeel et al. 43	1999	DIEP	S-W	ACB of ICN 10^th^ -12^th^ thoracic nerve	AB-LCB of 4^th^ ICN	increases the quality and quantity of sensation

S-W: Semmes-Weinstein monofilament, LFCN: the lateral femoral cutaneous nerve ACFN : anterior cutaneous branch of the femoral nerve LTP: Lateral Thigh Perforator

## Abbreviation

LDM: Latissimus dorsi
DIEP: Deep inferior epigastric perforator
TRAM: Transverse rectus abdominis muscle
LTP: Lateral Thigh Perforator
S-W: Semmes-Weinstein monofilament
PSSD: probabilistic species sensitivity distribution

## References

[1] Runowicz CD, Leach CR, Henry NL, Henry KS, Mackey HT, Cowens-Alvarado RL, Cannady RS, Pratt-Chapman ML, Edge SB, Jacobs LA (2016). American Cancer Society/American Society of Clinical Oncology Breast Cancer Survivorship Care Guideline. CA Cancer J Clin.

[2] Kostidou E, Schmelz M, Hasemaki N, Kokotis P (2019). Objective Methods for Breast Sensibility Testing. Plast Reconstr Surg.

[3] Pandya S, Moore RG (2011). Breast development and anatomy. Clin Obstet Gynecol.

[4] Rehnke RD, Groening RM, Van Buskirk ER, Clarke JM (2018). Anatomy of the Superficial Fascia System of the Breast: A Comprehensive Theory of Breast Fascial Anatomy. Plast Reconstr Surg.

[5] Bijkerk E, Cornelissen AJM, Sommer M, Van Der Hulst RRWJ, Lataster A, Tuinder SMH (2020). Intercostal nerve block of the anterior cutaneous branches and the sensibility of the female breast. Clin Anat.

[6] Zheng Y, Zhong M, Ni C, Yuan H, Zhang J (2017). Radiotherapy and nipple-areolar complex necrosis after nipple-sparing mastectomy: a systematic review and meta-analysis. Radiol Med.

[7] Riccio CA, Zeiderman MR, Chowdhry S, Brooks RM, Kelishadi SS, Tutela JP, Choo J, Yonick DV, Wilhelmi BJ (2015). Plastic Surgery of the Breast: Keeping the Nipple Sensitive. Eplasty.

[8] Sarhadi NS, Shaw-Dunn J, Soutar DS (1997). Nerve supply of the breast with special reference to the nipple and areola: Sir Astley Cooper revisited. Clin Anat.

[9] Longo B, Campanale A, Santanelli di Pompeo F (2014). Nipple-areola complex cutaneous sensitivity: a systematic approach to classification and breast volume. J Plast Reconstr Aesthet Surg.

[10] Peled AW, Amara D, Piper ML, Klassen AF, Tsangaris E, Pusic AL (2019). Development and Validation of a Nipple-Specific Scale for the BREAST-Q to Assess Patient-Reported Outcomes following Nipple-Sparing Mastectomy. Plast Reconstr Surg.

[11] Cornelissen AJM, Tuinder SMH, Heuts EM, van der Hulst RRWJ, Slatman J (2018). What does a breast feel like? A qualitative study among healthy women. BMC Womens Health.

[12] Bulut T, Tahta M, Sener U, Sener M (2018). Inter- and intra-tester reliability of sensibility testing in healthy individuals. J Plast Surg Hand Surg.

[13] Klein HJ, Fakin RM, Ducommun P, Giesen T, Giovanoli P, Calcagni M (2016). Evaluation of Cutaneous Spatial Resolution and Pressure Threshold Secondary to Digital Nerve Repair. Plast Reconstr Surg.

[14] Puonti HK, Broth TA, Soinila SO, Hallikainen HK, Jääskeläinen SK (2017). How to Assess Sensory Recovery After Breast Reconstruction Surgery?. Clin Breast Cancer.

[15] Karagoz H, Ozturk S, Siemionow M (2016). Comparison of Neurosensory Assessment Methods in Plastic Surgery. Ann Plast Surg.

[16] Beugels J, Bijkerk E, Lataster A, Heuts EM, van der Hulst RRWJ, Tuinder SMH (2021). Nerve Coaptation Improves the Sensory Recovery of the Breast in DIEP Flap Breast Reconstruction. Plast Reconstr Surg.

[17] Beugels J, Cornelissen AJM, van Kuijk SMJ, Lataster A, Heuts EM, Piatkowski A, Spiegel AJ, van der Hulst RRWJ, Tuinder SMH (2019). Sensory Recovery of the Breast following Innervated and Noninnervated DIEP Flap Breast Reconstruction. Plast Reconstr Surg.

[18] van Verschuer VMT, Mureau MAM, Gopie JP, Vos EL, Verhoef C, Menke-Pluijmers MBE, Koppert LB (2016). Patient Satisfaction and Nipple-Areola Sensitivity After Bilateral Prophylactic Mastectomy and Immediate Implant Breast Reconstruction in a High Breast Cancer Risk Population: Nipple-Sparing Mastectomy Versus Skin-Sparing Mastectomy. Ann Plast Surg.

[19] Yap LH, Whiten SC, Forster A, Stevenson HJ (2005). Sensory recovery in the sensate free transverse rectus abdominis myocutaneous flap. Plast Reconstr Surg.

[20] de Sá JZ, Lopes P, Santa-Cruz F, de Oliveira Rodrigues AE, Santos DM, de Andrade Aguiar JL (2021). Evaluation of Sensitivity in Specific Points of the Areola and Nipple of Patients Submitted to Reduction Mammoplasty With Periareolar Dermis Release: A Randomized Controlled Study. Aesthet Surg J.

[21] Bijkerk E, van Kuijk SMJ, Beugels J, Cornelissen AJM, Heuts EM, van der Hulst RRWJ, Tuinder SMH (2019). Breast sensibility after mastectomy and implant-based breast reconstruction. Breast Cancer Res Treat.

[22] Klasson S, Svensson K, Wollmer P, Velander P, Svensson H (2014). Blood flow dynamics and sensitivity in breasts after reconstruction with DIEP-flap. J Plast Surg Hand Surg.

[23] Schlenz I, Rigel S, Schemper M, Kuzbari R (2005). Alteration of nipple and areola sensitivity by reduction mammaplasty: a prospective comparison of five techniques. Plast Reconstr Surg.

[24] Momeni A, Meyer S, Shefren K, Januszyk M (2021). Flap Neurotization in Breast Reconstruction with Nerve Allografts: 1-year Clinical Outcomes. Plast Reconstr Surg Glob Open.

[25] Pek W-S, Tan B-K, Ru Ng YY, Kiak Mien Tan V, Rasheed MZ, Kiat Tee Tan B, Ong KW, Ong YS (2018). Immediate breast reconstruction following nipple-sparing mastectomy in an Asian population: Aesthetic outcomes and mitigating nipple-areolar complex necrosis. Arch Plast Surg.

[26] Godbout E, Farmer L, Bortoluzzi P, Caouette Laberge L (2013). Donor-site morbidity of the inferior gluteal artery perforator flap for breast reconstruction in teenagers. Can J Plast Surg.

[27] Hefter W, Elvenes OP, Lindholm P (2003). A retrospective quantitative assessment of breast sensation after lateral pedicle mammaplasty. Br J Plast Surg.

[28] Tevlin R, Brazio P, Tran N, Nguyen D (2021). Immediate targeted nipple-areolar complex re-innervation: Improving outcomes in immediate autologous breast reconstruction. J Plast Reconstr Aesthet Surg.

[29] Cornelissen AJM, Beugels J, van Kuijk SMJ, Heuts EM, Rozen SM, Spiegel AJ, van der Hulst RRWJ, Tuinder SMH (2018). Sensation of the autologous reconstructed breast improves quality of life: a pilot study. Breast Cancer Res Treat.

[30] Chiari A, Nunes TA, Grotting JC, Cotta FB, Gomes RCB (2012). Breast sensitivity before and after the L short-scar mammaplasty. Aesthetic Plast Surg.

[31] Hamdi M, Blondeel P, Van de Sijpe K, Van Landuyt K, Monstrey S (2003). Evaluation of nipple-areola complex sensitivity after the latero-central glandular pedicle technique in breast reduction. Br J Plast Surg.

[32] Beugels J, van Kuijk SMJ, Lataster A, van der Hulst RRWJ, Tuinder SMH (2021). Sensory Recovery of the Breast following Innervated and Noninnervated Lateral Thigh Perforator Flap Breast Reconstruction. Plast Reconstr Surg.

[33] Cornelissen AJM, Beugels J, Lataster A, Heuts EM, Rozen SM, Spiegel AJ, van der Hulst RRWJ, Tuinder SMH (2018). Comparing the sensation of common donor site regions for autologous breast reconstruction to that of a healthy breast. J Plast Reconstr Aesthet Surg.

[34] Mori H, Okazaki M (2011). Is the sensitivity of skin-sparing mastectomy or nipple-sparing mastectomy superior to conventional mastectomy with innervated flap?. Microsurgery.

[35] Mofid MM, Dellon AL, Elias JJ, Nahabedian MY (2002). Quantitation of breast sensibility following reduction mammaplasty: a comparison of inferior and medial pedicle techniques. Plast Reconstr Surg.

[36] Silhol T, Chaouat M, Noel W, Mimoun M, Boccara D (2021). Interindividual and Intraindividual Variations of Breast Sensitivity Before and After Breast Reduction: A Prospective Study. Ann Plast Surg.

[37] Heine N, Koch C, Brebant V, Kehrer A, Anker A, Prantl L (2017). Breast sensitivity after mastectomy and autologous reconstruction. Clin Hemorheol Microcirc.

[38] Puonti HK, Jääskeläinen SK, Hallikainen HK, Partanen TA (2011). A new approach to microneurovascular TRAM-flap breast reconstruction-a pilot study. J Plast Reconstr Aesthet Surg.

[39] Yano K, Hosokawa K, Takagi S, Nakai K, Kubo T (2002). Breast reconstruction using the sensate latissimus dorsi musculocutaneous flap. Plast Reconstr Surg.

[40] Bijkerk E, van Kuijk SMJ, Lataster A, van der Hulst RRWJ, Tuinder SMH (2020). Breast sensibility in bilateral autologous breast reconstruction with unilateral sensory nerve coaptation. Breast Cancer Res Treat.

[41] Puonti HK, Jääskeläinen SK, Hallikainen HK, Partanen TA (2017). Improved sensory recovery with a novel dual neurorrhaphy technique for breast reconstruction with free muscle sparing TRAM flap technique. Microsurgery.

[42] Tindholdt TT, Tønseth KA (2008). Spontaneous reinnervation of deep inferior epigastric artery perforator flaps after secondary breast reconstruction. Scand J Plast Reconstr Surg Hand Surg.

[43] Blondeel PN, Demuynck M, Mete D, Monstrey SJ, Van Landuyt K, Matton G, Vanderstraeten GG (1999). Sensory nerve repair in perforator flaps for autologous breast reconstruction: sensational or senseless?. Br J Plast Surg.

[44] Akdeniz Dogan Z, Farhadi J (2020). Evaluation of Sensation on Mastectomy Skin Flaps following Immediate Breast Reconstruction. J Reconstr Microsurg.

[45] Dossett LA, Lowe J, Sun W, Lee MC, Smith PD, Jacobsen PB, Laronga C (2016). Prospective evaluation of skin and nipple-areola sensation and patient satisfaction after nipple-sparing mastectomy. J Surg Oncol.

[46] Pitanguy I, Vaena M, Radwanski HN, Nunes D, Vargas AF (2007). Relative implant volume and sensibility alterations after breast augmentation. Aesthetic Plast Surg.

[47] Edsander-Nord A, Wickman M, Hansson P (1999). Somatosensory status after pedicled or free TRAM flap surgery: a retrospective study. Plast Reconstr Surg.

[48] de Sá JZ, Braga ACCR, Barreto RHC, Ramos A da S, de Oliveira Rodrigues AE, Santa-Cruz F, Aguiar JL de A (2020). Sensitivity of the Nipple-Areola Complex in Reduction Mammaplasty Following Periareolar Dermis Section. Aesthet Surg J.

[49] Brown T (2016). Objective Sensory Changes Following Subfascial Breast Augmentation. Aesthet Surg J.

[50] Temple CLF, Tse R, Bettger-Hahn M, MacDermid J, Gan BS, Ross DC (2006). Sensibility following innervated free TRAM flap for breast reconstruction. Plast Reconstr Surg.

[51] Place MJ, Song T, Hardesty RA, Hendricks DL (1997). Sensory reinnervation of autologous tissue TRAM flaps after breast reconstruction. Ann Plast Surg.

[52] Masgoret P, de Soto I, Caballero Á, Ríos J, Gomar C (2020). Incidence of contralateral neurosensitive changes and persistent postoperative pain 6 months after mastectomy: A prospective, observational investigation. Medicine (Baltimore).

[53] Stromps J-P, Bozkurt A, Grieb G, Kim B-S, Wiezik M, Pallua N (2016). Spontaneous Reinnervation of Deep Inferior Epigastric Perforator Flaps after Delayed Breast Reconstruction. J Reconstr Microsurg.

[54] Rodriguez-Unda NA, Bello RJ, Clarke-Pearson EM, Sanyal A, Cooney CM, Manahan MA, Rosson GD (2017). Nipple-Sparing Mastectomy Improves Long-Term Nipple But Not Skin Sensation After Breast Reconstruction: Quantification of Long-Term Sensation in Nipple Sparing Versus Non-nipple Sparing Mastectomy. Ann Plast Surg.

[55] Spiegel AJ, Menn ZK, Eldor L, Kaufman Y, Dellon AL (2013). Breast Reinnervation: DIEP Neurotization Using the Third Anterior Intercostal Nerve. Plast Reconstr Surg Glob Open.

[56] Longo B, Campanale A, Farcomeni A, Santanelli F (2013). Long-term sensory recovery of nipple-areola complex following superolateral pedicled reduction mammaplasty. Plast Reconstr Surg.

[57] Santanelli F, Longo B, Angelini M, Laporta R, Paolini G (2011). Prospective computerized analyses of sensibility in breast reconstruction with non-reinnervated DIEP flap. Plast Reconstr Surg.

[58] Dejean MF, Dabi Y, Goutard M, Taveau CB, Lantieri LA, Lellouch AG (2021). Deep inferior epigastric perforator free flap in elderly women for breast reconstruction: The experience of a tertiary referral center and a literature review. Breast J.

[59] Opsomer D, Vyncke T, Ryx M, Stillaert F, Van Landuyt K, Blondeel P (2020). Comparing the Lumbar and SGAP Flaps to the DIEP Flap Using the BREAST-Q. Plast Reconstr Surg.

[60] Temple CLF, Ross DC, Kim S, Tse R, Bettger-Hahn M, Gan BS, MacDermid J (2009). Sensibility following innervated free TRAM flap for breast reconstruction: Part II. Innervation improves patient-rated quality of life. Plast Reconstr Surg.

[61] Khan A, Zhang J, Sollazzo V, Mohammed K, Gui G (2016). Sensory change of the reconstructed breast envelope after skin-sparing mastectomy. Eur J Surg Oncol.

[62] Magarakis M, Venkat R, Dellon AL, Shridharani SM, Bellamy J, Vaca EE, Jeter SC, Zoras O, Manahan MA, Rosson GD (2013). Pilot study of breast sensation after breast reconstruction: evaluating the effects of radiation therapy and perforator flap neurotization on sensory recovery. Microsurgery.

[63] Louro J, Román M, Posso M, Comerma L, Vidal C, Saladié F, Alcantara R, Sanchez M, Quintana MJ, Del Riego J (2020). Differences in breast cancer risk after benign breast disease by type of screening diagnosis. Breast.

[64] Rosselet C, Zennou-Azogui Y, Escoffier G, Kirmaci F, Xerri C (2008). Experience-dependent changes in spatiotemporal properties of cutaneous inputs remodel somatosensory cortical maps following skin flap rotation. Eur J Neurosci.

[65] Zhang M, Shen G, Zhang S, Cui Z, Qian J (2017). Advantages of the modified double ring areolar incision over the traditional areolar incision in multicentric breast fibroadenoma surgery. Thorac Cancer.

[66] Wagner JL, Fearmonti R, Hunt KK, Hwang RF, Meric-Bernstam F, Kuerer HM, Bedrosian I, Crosby MA, Baumann DP, Ross MI (2012). Prospective evaluation of the nipple-areola complex sparing mastectomy for risk reduction and for early-stage breast cancer. Ann Surg Oncol.

[67] Muslu Ü, Demirez DŞ, Uslu A, Korkmaz MA, Filiz MB (2018). Comparison of Sensory Changes Following Superomedial and Inferior Pedicle Breast Reduction. Aesthetic Plast Surg.

[68] Silhol T, Chaouat M, Harizi R, Lambert J, Noel W, Mimoun M, Boccara D (2019). A prospective comparison of breast sensibility after reduction mammoplasty: Superior versus superomedial pedicle. J Plast Reconstr Aesthet Surg.

[69] Garcia ES, Veiga DF, Sabino-Neto M, Beraldo Cardoso FNM, Batista IO, Leme RM, Cabral IV, Novo NF, Ferreira LM (2015). Sensitivity of the Nipple-Areola Complex and Sexual Function Following Reduction Mammaplasty. Aesthet Surg J.

[70] Chiummariello S, Angelisanti M, Arleo S, Alfano C (2013). Evaluation of the sensitivity after reduction mammoplasty. Our experience and review of the literature. Ann Ital Chir.

[71] Greco R, Noone B (2017). Evidence-Based Medicine: Reduction Mammaplasty. Plast Reconstr Surg.

[72] Schulz S, Zeiderman MR, Gunn JS, Riccio CA, Chowdhry S, Brooks R, Choo JH, Wilhelmi BJ (2017). Safe Plastic Surgery of the Breast II: Saving Nipple Sensation. Eplasty.

[73] Schratt J, Binter A, Rab M (2014). [Reduction mammaplasty with a superior pedicle - a retrospective 10-year follow-up analysis of 33 patients]. Handchir Mikrochir Plast Chir.

[74] Sensitivity of the nipple-areola complex and areolar pain following aesthetic breast augmentation in a retrospective series of 1200 patients periareolar versus submammary incision - PubMed. Available at https://pubmed.ncbi.nlm.nih.gov/21921773/.

[76] Faulkner HR, Colwell AS, Liao EC, Winograd JM, Austen WG (2016). Thermal Injury to Reconstructed Breasts from Commonly Used Warming Devices: A Risk for Reconstructive Failure. Plast Reconstr Surg Glob Open.

[77] Ilonzo N, Tsang A, Tsantes S, Estabrook A, Thu Ma AM (2017). Breast reconstruction after mastectomy: A ten-year analysis of trends and immediate postoperative outcomes. Breast.

[78] Chirappapha P, Srichan P, Lertsithichai P, Thaweepworadej P, Sukarayothin T, Leesombatpaiboon M, Kongdan Y (2018). Nipple-Areola Complex Sensation after Nipple-sparing Mastectomy. Plast Reconstr Surg Glob Open.

[79] Wang Y, Wu J-X, Guan S (2017). A Technique of Endoscopic Nipple-Sparing Mastectomy for Breast Cancer. JSLS.

[80] Isenberg JS, Spinelli H (2004). Further experience with innervated autologous flaps in postoncologic breast reconstruction. Ann Plast Surg.

[81] Isenberg JS (2002). Sense and sensibility: breast reconstruction with innervated TRAM flaps. J Reconstr Microsurg.

[82] Blondeel PN (1999). The sensate free superior gluteal artery perforator (S-GAP) flap: a valuable alternative in autologous breast reconstruction. Br J Plast Surg.

[83] Lee JH, Ahn HC, Chung MS (2015). Herpes zoster in a free transverse rectus abdominis myocutaneous flap after delayed breast reconstruction: evidence of spontaneous reinnervation. Ann Plast Surg.

[84] Ducic I, Yoon J, Momeni A, Ahcan U (2018). Anatomical Considerations to Optimize Sensory Recovery in Breast Neurotization with Allograft. Plast Reconstr Surg Glob Open.

[85] Mericli AF, Szpalski C, Schaverien MV, Selber JC, Adelman DM, Garvey PB, Villa MT, Robb G, Baumann DP (2019). The Latissimus Dorsi Myocutaneous Flap Is a Safe and Effective Method of Partial Breast Reconstruction in the Setting of Breast-Conserving Therapy. Plast Reconstr Surg.

[86] Szychta P, Butterworth M, Dixon M, Kulkarni D, Stewart K, Raine C (2013). Breast reconstruction with the denervated latissimus dorsi musculocutaneous flap. Breast.

[87] Beugels J, Cornelissen AJM, Spiegel AJ, Heuts EM, Piatkowski A, van der Hulst RRWJ, Tuinder SMH (2017). Sensory recovery of the breast after innervated and non-innervated autologous breast reconstructions: A systematic review. J Plast Reconstr Aesthet Surg.

[88] Peled AW, Peled ZM (2019). Nerve Preservation and Allografting for Sensory Innervation Following Immediate Implant Breast Reconstruction. Plast Reconstr Surg Glob Open.

[89] Chen G, Zhang Y, Xue J, Zhu X, Liu C, Sun L, Gu X, Zhang H, Liu C (2019). Surgical Outcomes of Implant-based Breast Reconstruction Using TiLoop Bra Mesh Combined With Pectoralis Major Disconnection. Ann Plast Surg.

[90] EBCTCG (Early Breast Cancer Trialists' Collaborative Group), McGale P, Taylor C, Correa C, Cutter D, Duane F, Ewertz M, Gray R, Mannu G, Peto R (2014). Effect of radiotherapy after mastectomy and axillary surgery on 10-year recurrence and 20-year breast cancer mortality: meta-analysis of individual patient data for 8135 women in 22 randomised trials. Lancet.

[91] Admoun C, Mayrovitz H (2021). Choosing Mastectomy vs. Lumpectomy-With-Radiation: Experiences of Breast Cancer Survivors. Cureus.

[92] Lam E, Wong G, Zhang L, Drost L, Karam I, Yee C, McCurdy-Franks E, Razvi Y, Ariello K, Wan BA (2021). Self-reported pain in breast cancer patients receiving adjuvant radiotherapy. Support Care Cancer.

[93] Andersen KG, Gärtner R, Kroman N, Flyger H, Kehlet H (2012). Persistent pain after targeted intraoperative radiotherapy (TARGIT) or external breast radiotherapy for breast cancer: a randomized trial. Breast.

